# Resection-reconstruction arthroplasty for giant cell tumor of distal radius

**DOI:** 10.4103/0019-5413.65134

**Published:** 2010

**Authors:** Kabul C Saikia, Munin Borgohain, Sanjeev K Bhuyan, Sanjiv Goswami, Anjan Bora, Firoz Ahmed

**Affiliations:** Department of Orthopedics, Gauhati Medical College, Guwahati, India

**Keywords:** Distal radius, giant cell tumor, resection reconstruction

## Abstract

**Background:**

Giant cell tumor (GCT) of the distal radius poses problems for reconstruction after resection. Several reconstructive procedures like vascularized and non-vascularized fibular graft, osteo-articular allograft, ceramic prosthesis and megaprosthesis are in use for substitution of the defect in the distal radius following resection. Most authors advocate wrist arthrodesis following resection of distal radius and non vascularized fibular graft. Here we have analyzed the results of aggressive benign GCTs of the distal radius treated by resection and reconstruction arthroplasty using autogenous non-vascularized fibular graft.

**Materials and Methods:**

Twenty-four cases of giant cell tumor of the distal radius (mean age 32 years, mean follow-up 6.6 years) treated by en-bloc resection and reconstruction arthroplasty using autogenous non-vascularized ipsilateral fibular graft with a minimum followup of two years have been included in this retrospective study. Nineteen cases were of Campanacci grade III and five were of Grade II recurrence. The mean resected length of the radius was 9.5 (8-12) cm. Routine radiographs and clinical assessments regarding pain, instability, recurrence, hand grip strength and functional status were done at regular intervals and functional results were assessed using (musculoskeletal tumor society) MSTS-87 scoring.

**Results:**

Early radiological union at host-graft junction was achieved at mean 12.5 weeks, (range 12-14 weeks) and solid incorporation with callus formation was observed in mean 29 weeks (range 28-32 weeks) in all the cases. Satisfactory range of motion (mean 63%, range 52-78%) of the wrist was achieved in 18 cases. Grip strength compared to the contralateral hand was found to be 67% (range 58-74%). Functional results were excellent in six cases (25%), good in 14 cases (58.3%) and four (16.7%) cases had fair results. Soft tissue recurrence was seen in one patient. The most commonly encountered complication was fibulo-carpal subluxation (10 cases, 41.7%).

**Conclusion:**

Resection of the distal radius and reconstruction arthroplasty with non-vascularized proximal fibular graft is useful in preserving the functional movement and stability of the wrist as well as achieving satisfactory range of movement and grip strength.

## INTRODUCTION

Giant cell tumor (GCT) of bone is a benign but locally aggressive tumor with tendency for local recurrence.^1^ Absence of absolute clinical, radiological or histological parameters renders the tendency of any lesion to recur or metastasize. Distal radius is the third most commonly involved site of skeletal GCTs (10% cases) next to distal femur and proximal tibia.^2^,^3^ Many methods have been advocated for the management of distal radial GCTs. Goals of treatment are to achieve satisfactory removal of the tumor, lessen the chance of local recurrence and to preserve as much wrist function as possible. The treatment consists of either curettage or en-bloc resection of the lesion with subsequent reconstructions.^1^,^4^,^5^,^6^ Though curettage and bone grafting can preserve joint functions, it has been associated with high local recurrence rate of 27% to 54%.^7^–^10^ Walthar (1911) was the first to describe the use of a free non vascular proximal fibular graft to replace the resected distal radius.^11^ Most of the authors have reported various success rates with the procedure. 11–17 The use of free vascularized proximal fibular graft has produced encouraging results.^18^–^20^

The purpose of this study is to analyze the results of GCT of the distal radius (Campanacci grade III and recurrent grade II) treated by resection and reconstruction arthroplasty using autogenous non-vascularized fibular graft.

## MATERIALS AND METHODS

Twenty-four cases of GCT involving the distal radius operated by en-bloc resection of tumor followed by reconstruction of the gap with autogenous non-vascularized fibular graft with a minimum 2 years followup are included in this retrospective study. They were operated by the at least one out of the 3 senior authors (KS, SKB, MB). The cases were operated from January 1993 to March 2007. Informed consent was obtained from all the patients. The study was authorized by the ethics committee of our institute and was performed in accordance with the Ethical standards of the 1975 declaration of Helsinki, revised in 2000. The mean follow-up was 6.6 years (2-11 years). Nineteen cases were Campanacci Grade III and five were Grade II recurrent GCTs following 5,8,18,12,3 months respectively (mean 9.2) treated earlier with curettage and bone grafting/cementing. Confirmation of diagnosis was by needle aspiration cytology in 14 cases (58.3%) and open biopsy in 10 cases (41.7%).

Criterion for inclusion was evidence is radiographic features characteristic of GCT on roentgenogram, CT scan or MRI and confirmation by needle aspiration cytology or open biopsy [Figure 1A]. The cases included in the study were primarily treated in our institute and have been followed up for a minimum period of two years. Using a volar or dorsal approach, depending upon anterior(n=15) or posterior expansion(n=9) of the lesion, the tumor along with biopsy scar was resected. Four to five cm of distal radius was excised along with the tumor as safe margin in all the cases. Clearance biopsies taken from the safe margins were negative in all the cases. The bone defect after excision of the tumor ranged from eight to twelve (mean 9.5) cm. Care was taken to preserve the neurovascular bundle while resecting the tumor mass from the surrounding soft tissue. Reconstruction of the bony defect was done using ipsilateral proximal fibular transplant.

**Figure 1A:**
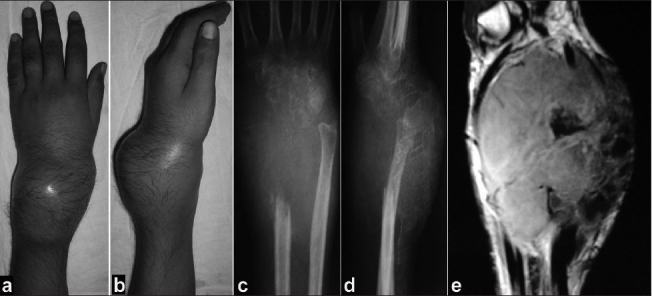
Clinical photograph dorsal (a) and side (b) views, with giant cell tumour of the lower end of radius. Anteroposterior (c) and lateral (d) radiograph (e) saggital MRI showing extensive bony destuction campanacci grade III giant cell tumour

The articular surface of the head of the fibula was placed over the scapho-lunate articular surface and fixed to the carpals with one or two 1.5 mm K-wires inserted obliquely. The excised end of the radius and the transplant were fixed with a small DCP taking 12 cortices fixation. The lateral ligament of the wrist was created by attaching the part of the fibular collateral ligament. Another K-wire was inserted transversely to stabilize the newly created fibulo-ulnar joint [Figure 1B, a]. Iliac cancellous bone grafting was done routinely at the host-graft junction. The limb was immobilized in a long arm cast for 8-10 weeks and followed by a forearm brace after removing the K-wires for mobilization. Protected brace was continued for another 14-16 weeks until solid union was achieved radiologically [Figure 1B, b]. Routine radiographs were taken at three monthly intervals for one year and thereafter every six months to exclude local recurrence.

**Figure 1B:**
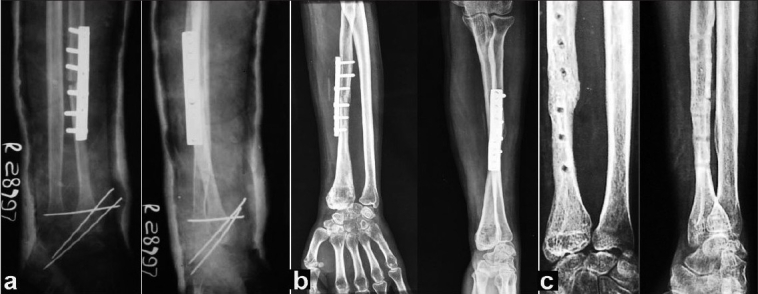
X-ray anteroposterior and lateral views of same patient (a) immediate postoperative X-Ray showing plate and K-wire in position. (b) 18 months postoperative X-Ray showing sound union at host graft junction. (c) 6 years postoperative X-Ray after plate removal showing union at host graft junction

Clinical assessments regarding pain, instability, recurrence, hand grip strength and functional status were done at regular intervals of three-six months. The range of movement at wrist joint was measured with a goniometer and grip strength was assessed in comparison with the opposite hand. Functional results were evaluated by one of the senior authors (KS, SKB or MB) using Musculoskeletal Tumor Society (MSTS 87) rating for limb salvage.^21^

## RESULTS

The mean age of 14 male and 10 female patients included in the study was 32 years (17-56 years). Maximum number of cases (n=12) were in the age group of 21-30 years. The side affected was left in 14 and right in 10 cases. Two of the five patients of Grade II recurrent tumors were earlier treated with curettage and bone cementing and the rest of them had curettage and bone grafting. Average operating time was 1.5 hours. The mean resected length of the radius was 9.5 (range 8-12) cm.

Early radiological union at host graft junction was achieved at 12-14 weeks (mean 12.5 weeks) and solid incorporation with callus formation was seen in 28-32 weeks (mean 29) in all the cases [Figure 1B, c].

The average range of motion of the involved wrist was 50° dorsiflexion, 38° palmar flexion, 12° radial deviation, 22° ulnar deviation, 52° supination and 46° pronation. They retained 63% of contralateral range of wrist motion. Grip strength (by Dynamometer) compared to the contralateral hand was found to be 58-74% (average 67%). Functional results were excellent in six cases, good in 14 cases and four cases had fair results. Details relating to patients’ data and functional results are mentioned in Table 1.

**Table 1:** Clinical details of 24 patients of resection-reconstruction arthroplasty of GCT of distal radius and Functional results*

Age	Sex	Side	Duration of follow-up (years)	Campanaccigrade	Grip strength (%)	Range of motion (%)	Functional rating	Complications	Remarks
25	Fm	R	4.0	3	68	63	G	-	-
17	M	L	6.0	3	72	74	E	-	-
30	M	L	7.4	2 recurr	69	62	G	-	-
36	Fm	L	2.0	3	66	60	G	Infection	Healed with curettage and antibiotics
28	M	R	5.0	3	66	61	G	Mild subluxation with fibulocarpal diastasis	-
20	M	L	7.0	3	74	70	E	-	-
56	M	L	3.6	2 recurr	68	74	E	-	-
29	Fm	R	6.0	3	60	52	F	Fibulo carpal arthrosis	-
28	M	R	8.0	2 recurr	68	60	G	-	-
48	Fm	L	7.2	3	58	52	F	Subluxated wrist	-
27	M	R	11.0	3	72	71	E	-	-
33	M	L	9.0	3	65	62	G	-	-
29	Fm	R	5.8	3	68	60	G	Soft tissue recurrence	Resected
18	M	L	10.4	3	69	62	G	-	-
31	Fm	L	7.5	3	58	53	F	Fibulo carpal arthrosis	-
37	Fm	L	2.2	3	74	75	E	-	-
27	M	R	5.2	2 recurr	66	63	G	Graft fracture	United after 10 weeks of immobilization
21	M	L	8.3	3	68	62	G	-	-
55	M	L	3.5	3	66	62	G	Mild subluxation with fibulocarpal diastasis	-
30	Fm	R	6.4	3	56	52	F	Subluxated wrist	-
27	M	R	10.2	2 recurr	67	62	G	-	-
49	Fm	L	7.0	3	76	78	E	-	-
28	Fm	R	7.4	3	68	60	G	-	-
39	M	L	8.2	3	66	62	G	-	-

* Musculo Skeletal Tumour Society -87 scoring

M = Male; R = Right; E = Excellent; Recurr = Recurrence; Fm = Female; L = Left; G = Good; F = Fair

One patient (Case no. 4) who developed infection was treated by thorough curettage and appropriate antibiotics (cefuroxime). Infection subsided in three months time. Another case of soft tissue recurrence without any bony involvement (Case no. 13) was treated with total excision of the mass. No further recurrence is seen in this case till the most recent follow up. One patient had graft fracture (Case no. 17) which united after immobilization in a cast for 10 weeks.

### Subluxation

**Figure 2:**
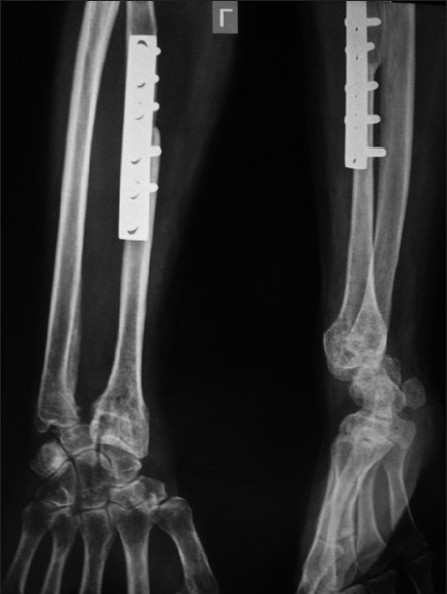
X-ray – Anteroposterior and lateral views showing moderate subluxation of fibulo-carpal joint with ulno-fibula diastasis

There was significant subluxation of the carpals over the transplanted fibula in two patients (Case nos. 10, 20) resulting in pain, deformity and loss of function. In another six cases (25%), mild to moderate subluxation was observed radiologically but the wrist was pain free with normal appearance at recent follow-up. It did not affect the function significantly. The subluxation occurred between 3-12 months after surgery in 7 out of eight cases. Another two cases (Case nos. 5, 19) had moderate subluxation with diastasis between fibular head and distal ulna with intermittent mild pain [Figure 2]. They were relieved with wrist support brace. Two cases (Case nos. 8, 15) developed arthrosis of the fibulo-carpal joint without requiring any secondary procedure.

### Donor site morbidity

At the donor site of proximal fibula, no instability of the knee was detected at operation. Peroneal nerve palsy occurred in the donor limb in two cases, but recovered spontaneously at six weeks and 10 weeks respectively. None of the patients reported with pulmonary metastasis at the time of presentation or on subsequent follow-up.

## DISCUSSION

Giant cell tumor is a challenge for the surgeons both for cure and rehabilitation. Most patients with GCT are young with normal life expectancy. The aim of treatment is to remove the tumor, reduce the chances of recurrence and preserve the joint function. The defect created by the excision of the distal radius can be filled by non-vascularized autogenous proximal fibular graft,^13^–^17^ vascularized fibula,^18^,^19^ or vascularized pedicle graft of the ulna.^20^,^22^

Local recurrence and loss of joint function are still major problems following surgery. Bone grafting or bone cementing after intralesional curettage of the tumor has high local recurrence rate. Many authors reported that GCT of distal radius is particularly aggressive and has a high rate of local recurrence.^8^,^10^,^23^,^24^ Eckardt *et al*. recommended en-bloc resection for most grade III lesions.^1^ Wide resection of the distal radius has been recommended to treat Grade III GCT when the tumor breaks through the cortex on dorsal and volar sides, when tumor invades the wrist joint or more than 50% of the surrounding metaphysis has been destroyed.^24^,^25^ In our study also, we have followed this recommendation.

Resection of distal radius and reconstruction with autogenous non-vascularized ipsilateral fibula offers several advantages like more congruency of carpal joint, rapid incorporation as autograft and easy accessibility without significant donor site morbidity. Structural change is also minimal. Moreover, immunogenic reactions are absent and bone banking facilities or graft matching procedures are not required. No allograft was used in our study. The high cost involved and the absence of a proper bone bank prohibits us from using allograft in our setup. Case reports of joint preservation using vascularized fibula or prosthesis are found to be few and inconclusive.^18^,^22^ Vascularized fibular grafting has been reported to speed up the healing at host-graft junction thereby reducing the period of immobilization required. The operating time for vascularized fibular graft often reaches 12-14 hours and requires sacrifice of two major vessels. Dissection to obtain the fibula and its vascular pedicle and the isolation of its recipient vessels requires meticulous attention.^18^ Sophisticated infrastructure, skill and prolonged operating time have made its use limited.

The most commonly encountered complication in our series is found to be fibulo-carpal subluxation. Two of our cases developed a subluxated wrist with pain and partial loss of function. Another six cases had radiological subluxation of the wrist and two cases had diastasis of the fibulo-ulnar joint. These patients were clinically asymptomatic and the radiographic findings were found during routine periodic check-up. Wrist function in these patients was good with little limitation in daily activities. In a series of 6 patients, Cheng *et al*. reported two patients with diastasis of the distal fibulo-ulnar joint.^24^ Dhammi *et al*. reported 10 cases (n=16) of wrist subluxation.^26^ Lackman *et al*. reported one case each of volar displacement and radial deviation (n=12).^27^ Saraf *et al*. reported significant subluxation of wrist in two cases out of 15 treated with plate fixation resulting in significant pain, deformity and loss of function.^28^ A proper length of fibular graft is a must to maintain the radial height and to prevent subluxation of the wrist joint. We ensured this by harvesting the fibula 2-3 mm more than the required length, which is the resected tumor length plus the safe margin. This 2-3 mm allowed us to achieve compression at the host-graft junction during fixation with DCP. A longer fibular graft will lead to subluxation of the wrist. K-wire fixation through the carpals and reconstruction of the lateral ligaments of wrist joint helps in stabilization of the wrist joint. Transverse fixation of the fibula-ulnar joint further helps in stabilization. Wrist stability is assured by fibrosis after 8-10 weeks following K-wire fixation. There is a chance of stiffness of the wrist with relatively longer duration of immobilization and consequently decrease in the hand grip strength.

We had only one recurrence (4.2%), that too, involving soft tissue. The lesion was treated with wide excision of the soft tissue mass. Murray *et al*. reported recurrences in 5 patients (n=18) of which 3 involved soft tissues only.^16^ There was no local recurrence reported by Cheng *et al*.^24^ The possible reason for this absence of recurrence was very few patients (n=6) and too short a follow-up to rule out late recurrences.^24^

Five cases of nonunions at the host graft junction were reported by Murray *et al*. in their series of 18 cases of distal radius GCT.^16^ This was attributed to inadequate fixation of the grafted fibula. Dhammi *et al*. reported five nonunions (n=16), while Lackman *et al*. reported 2 nonunions (n=12), all of which required secondary procedures.^26^,^27^ There were five cases of nonunions (n=15) in the series of Saraf *et al*. of which in two cases the limb was amputated due to recurrence.^28^ None of our cases had delayed union or nonunion. Twelve cortices’ fixation at the host-graft junction with a small DCP permits rigid fixation. In our series, intramedullary nail was not used for fixation of the fibular graft to the radial stump as compression cannot be achieved at the host graft junction, thereby increasing the chance of delayed or failure of union. Routine cancellous bone grafting was done at the host-graft junction, which prevents delayed union.^24^ There were graft fracture in 3 cases (n=18) in the Murray *et al*. series.^16^ We had graft fracture in only one case (4.2%). Cheng *et al*. did not report any graft fracture.^24^ Murray *et al*. had found donor site morbidity to be frequent like pain, weakness, lateral instability and transient peroneal nerve palsy.^16^ We had two cases of peroneal nerve palsy which recovered spontaneously by six and 10 weeks respectively. Lackman *et al*. did not report any donor site morbidity.^27^

Arthroplasty over arthrodesis was preferred in our study to retain joint mobility. We preserved an average of 63% (range 52-78%) of the contralateral range of wrist motion. The grip strength compared to the contralateral hand was found to be 67% (range 58-74%). Many authors reported good results (score around 74%) with arthrodesis for distal radius GCT.^16^,^29^ Wrist arthrodesis with autogenous fibula or ulna has been used after resection. An arthrodesis produces a painless, stable wrist, though at the expense of mobility. An arthrodesis reduces the functional score with minimal disability.^30^ Partial wrist arthrodesis has advantages over total arthrodesis.^31^

In patients who do not put high loads on the wrist, osteoarticular allografts may be a good option.^32^ Hatano *et al*. reported good results with two cases of a ceramic prosthesic replacement of distal radius with more than 10 years of follow-up.^33^ Bipolar hinged megaprosthesis was used by Natarajan *et al*. for GCT of distal radius.^34^ The 10-year prosthesis survival rate was 87.5%. Infection was the commonest complication. Ilizarov reconstruction of the distal radius after reconstruction of GCT has also been reported.^35^

GCT of the distal radius is best treated with excision of the distal radius and reconstruction by non-vascularized fibula with good functional results.^13^–^17^ Asavamongkolkul *et al*. reported good and excellent functional results in all seven patients of non-vascularized autogenous fibular graft reconstruction.^36^ Our method of resection and reconstruction with non-vascularized fibular graft, internal fixation with DCP with primary bone grafting, use of stabilizing K-wires across the newly formed wrist joint and ligament reconstruction has been advocated by many other authors.^12^,^26^,^28^

## CONCLUSION

Resection of distal radius and reconstruction with proximal fibular transplant is useful to preserve the functional movement and stability with normal appearance of the wrist. Further, this procedure eliminates the need for microvascular surgery. Our results showing satisfactory range of movements (63%) and sufficient grip strength (67%) with good functional results justify this procedure of reconstruction arthroplasty in case of Giant cell tumors of distal radius.
